# The Forest Observation System, building a global reference dataset for remote sensing of forest biomass

**DOI:** 10.1038/s41597-019-0196-1

**Published:** 2019-10-10

**Authors:** Dmitry Schepaschenko, Jérôme Chave, Oliver L. Phillips, Simon L. Lewis, Stuart J. Davies, Maxime Réjou-Méchain, Plinio Sist, Klaus Scipal, Christoph Perger, Bruno Herault, Nicolas Labrière, Florian Hofhansl, Kofi Affum-Baffoe, Alexei Aleinikov, Alfonso Alonso, Christian Amani, Alejandro Araujo-Murakami, John Armston, Luzmila Arroyo, Nataly Ascarrunz, Celso Azevedo, Timothy Baker, Radomir Bałazy, Caroline Bedeau, Nicholas Berry, Andrii M. Bilous, Svitlana Yu. Bilous, Pulchérie Bissiengou, Lilian Blanc, Kapitolina S. Bobkova, Tatyana Braslavskaya, Roel Brienen, David F. R. P. Burslem, Richard Condit, Aida Cuni-Sanchez, Dilshad Danilina, Dennis del Castillo Torres, Géraldine Derroire, Laurent Descroix, Eleneide Doff Sotta, Marcus V. N. d’Oliveira, Christopher Dresel, Terry Erwin, Mikhail D. Evdokimenko, Jan Falck, Ted R. Feldpausch, Ernest G. Foli, Robin Foster, Steffen Fritz, Antonio Damian Garcia-Abril, Aleksey Gornov, Maria Gornova, Ernest Gothard-Bassébé, Sylvie Gourlet-Fleury, Marcelino Guedes, Keith C. Hamer, Farida Herry Susanty, Niro Higuchi, Eurídice N. Honorio Coronado, Wannes Hubau, Stephen Hubbell, Ulrik Ilstedt, Viktor V. Ivanov, Milton Kanashiro, Anders Karlsson, Viktor N. Karminov, Timothy Killeen, Jean-Claude Konan Koffi, Maria Konovalova, Florian Kraxner, Jan Krejza, Haruni Krisnawati, Leonid V. Krivobokov, Mikhail A. Kuznetsov, Ivan Lakyda, Petro I. Lakyda, Juan Carlos Licona, Richard M. Lucas, Natalia Lukina, Daniel Lussetti, Yadvinder Malhi, José Antonio Manzanera, Beatriz Marimon, Ben Hur Marimon Junior, Rodolfo Vasquez Martinez, Olga V. Martynenko, Maksym Matsala, Raisa K. Matyashuk, Lucas Mazzei, Hervé Memiaghe, Casimiro Mendoza, Abel Monteagudo Mendoza, Olga V. Moroziuk, Liudmila Mukhortova, Samsudin Musa, Dina I. Nazimova, Toshinori Okuda, Luis Claudio Oliveira, Petr V. Ontikov, Andrey F. Osipov, Stephan Pietsch, Maureen Playfair, John Poulsen, Vladimir G. Radchenko, Kenneth Rodney, Andes H. Rozak, Ademir Ruschel, Ervan Rutishauser, Linda See, Maria Shchepashchenko, Nikolay Shevchenko, Anatoly Shvidenko, Marcos Silveira, James Singh, Bonaventure Sonké, Cintia Souza, Krzysztof Stereńczak, Leonid Stonozhenko, Martin J P Sullivan, Justyna Szatniewska, Hermann Taedoumg, Hans ter Steege, Elena Tikhonova, Marisol Toledo, Olga V. Trefilova, Ruben Valbuena, Luis Valenzuela Gamarra, Sergey Vasiliev, Estella F. Vedrova, Sergey V. Verhovets, Edson Vidal, Nadezhda A. Vladimirova, Jason Vleminckx, Vincent A. Vos, Foma K. Vozmitel, Wolfgang Wanek, Thales A. P. West, Hannsjorg Woell, John T. Woods, Verginia Wortel, Toshihiro Yamada, Zamah Shari Nur Hajar, Irié Casimir Zo-Bi

**Affiliations:** 1 Ecosystems Services and Management Program, International Institute for Applied Systems Analysis, Laxenburg, A-2361 Austria; 2 Forestry faculty, Bauman Moscow State Technical University, Mytischi, 141005 Russia; 3 Laboratoire Evolution et Diversité Biologique CNRS/Université Paul Sabatier, Toulouse, France; 4 School of Geography, University of Leeds, Leeds, LS2 9JT UK; 5 University College London, 30 Guilford Street, London, WC1N 1EH UK; 6 Forest Global Earth Observatory, Smithsonian Tropical Research Institute, P.O. Box 37012, Washington 20013, USA; 7 AMAP, IRD, CNRS, CIRAD, INRA, University Montpellier, Montpellier, France; 8 CIRAD, Forêts et Sociétés, Campus International de Baillarguet, Montpellier, F-34398 France; 9 Forêts et Sociétés, Univ Montpellier, CIRAD, Montpellier, F-34398 France; 10 European Space Agency, ESTEC, Noordwijk, The Netherlands; 11 Spatial Focus GmbH, Vienna, Austria; 12 Mensuration Unit, Forestry Commission of Ghana, 4 Third Avenue Ridge, Kumasi, POB M434 Ghana; 13 Center of Forest Ecology and Productivity of the Russian Academy of Sciences, Profsoyuznaya 84/32/14, Moscow, 117997 Russia; 14 Smithsonian Conservation Biology Institute, 1100 Jefferson Dr SW, Suite 3123, Washington, DC 20560-0705 USA; 15 Centre for International Forestry Research, CIFOR, Jalan CIFOR, Situ Gede, Bogor, 16115 Indonesia; 16 Universidad Autonoma Gabriel Rene Moreno, Santa Cruz, Bolivia; 17 Department of Geographical Sciences, University of Maryland, 2181 Lefrak Hall, College Park, MD 20742 USA; 18 Joint Remote Sensing Research Program, School of Earth and Environmental Sciences, University of Queensland, Chamberlain Building (35), Campbell Road, St Lucia Campus, Brisbane, 4072 Australia; 19 Museo de Historia Natural Noel Kempff Mercado, Universidad Autónoma Gabriel Rene Moreno Av. Irala 565 - casilla, 2489 Santa Cruz, Bolivia; 20 IBIF, Instituto Boliviano de Investigacion Forestal, Av. 6 de agosto # 28, Km 14 doble via La Guardia, Santa Cruz, Casilla 6204 Bolivia; 21 Embrapa, Rodovia AM 10, km 29, Manaus, AM 69010-970 Brazil; 22 Forest Research Institute, Department of Geomatics, Braci Leśnej 3, Sękocin Stary, Raszyn 05-090 Poland; 23 Naturalis Biodiversity Center, Leiden, The Netherlands; 24 ONF, ONF-Réserve de Montabo Cayenne Cedex, Cayenne, BP 7002; 97307 French Guiana; 25 The Landscapes and Livelihoods Group, 20 Chambers St, Edinburgh, EH1 1JZ UK; 26 National University of Life and Environmental Sciences of Ukraine, General Rodimtsev 19, Kyiv, 3041 Ukraine; 27 Herbier National du Gabon (IPHAMETRA), B.P 1165 Libreville, Gabon; 28 Institute of Biology, Komi Scientific Center, Ural Branch of Russian Academy of Sciences, Kommunisticheskaya 28, Syktyvkar, 167982 Russia; 29 School of Biological Sciences, University of Aberdeen, Cruickshank Building, St Machar Drive, Aberdeen, AB24 3UU UK; 30 Smithsonian Tropical Research Institute, Balboa, Ancon Panama 3092 Panama; 31 Department of Environment and Geography, University of York, Heslington, York YO10 5NG UK; 32 V.N. Sukachev Institute of Forest, Siberian Branch of the Russian Academy of Science, Academgorodok 50(28), Krasnoyarsk, 660036 Russia; 33 Instituto de Investigaciones de la Amazonía Peruana, Av. Abelardo Quiñones km 2.5, Iquitos, Apartado Postal 784 Peru; 34 U Gent-Woodlab, Laboratory of Wood Technology, Department of Environment, Ghent University, Ghent, 9000 Belgium; 35 CIRAD, UMR EcoFoG, Campus Agronomique - BP 701, Kourou, 97387 France French Guiana; 36 Embrapa, Rodovia Juscelino Kubitscheck, Km 5, no 2.600, Macapa, Caixa Postal 10, CEP: 68903-419 Brazil; 37 Embrapa, BR 364, Caixa postal 321, Rio Branco, CEP 69.900-970 Brazil; 38 Morton Arboretum, 4100 Illinois Rte. 53, Lisle, 60532 IL USA; 39 SI Entomology, Smithsonian Institution, PO Box 37012, MRC 187, Washington, DC DC 20013-7012 USA; 40 Department Forest Ecology and Management, The Swedish University of Agricultural Sciences, SLU, Umeå, SE-901 83 Sweden; 41 Geography, College of Life and Environmental Sciences, University of Exeter,Laver Building, North Park Road, Exeter, EX4 4QE UK; 42 Forestry Research Institute of Ghana, UP Box 63, KNUST, Kumasi, Ghana; 43 The Field Musium, 1400S Lake Shore Dr, Chicago, IL 60605 USA; 44 Universidad Politecnica de Madrid, Calle Ramiro de Maeztu, 7, Madrid, 28040 Spain; 45 Institut Centrafricain de Recherche Agronomique, ICRA, BP 122, Bangui, Central African Republic; 46 School of Biology, University of Leeds, Leeds, LS2 9JT UK; 47 FOERDIA, Forestry and Environment Research Development and Innovation Agency, Jalan Gunung Batu No 5, Bogor, 16610 Indonesia; 48 Instituto Nacional de Pesquisas da Amazônia - Coordenação de Pesquisas em Silvicultura Tropical, Manaus, 69060-001 Brazil; 49 Department of Ecology and Evolutionary Biology, University of California, 621 Charles E. Young Dr. South, Los Angeles, CA 90095-1606 USA; 50 Embrapa Amazonia Oriental, Travessa Doutor Enéas Pinheiro, Belém, PA 66095-903 Brazil; 51 World Wildlife Fund, Calle Diego de Mendoza 299, Santa Cruz de la Sierra, Bolivia; 52 Sodefor, boulevard François Mitterrand, Cocody, Abidjan, 01BP 3770 Côte d’Ivoire; 53 Global Change Research Institute CAS, Bělidla 986/4a, Brno, 603 00 Czech Republic; 54 Department of Geography and Earth Sciences, Aberystwyth University, Aberystwyth, SY23 3DB UK; 55 School of Geography and the Environment, University of Oxford, Oxford, OX1 3QY UK; 56 Laboratório de Ecologia Vegetal, Universidade do Estado de Mato Grosso, UNEMAT, Campus de Nova Xavantina, Nova Xavantina, Mato Grosso, 78.690-000 Brazil; 57 Jardín Botánico de Missouri; Universidad Nacional de San Antonio Abad del Cusco, Oxapampa, Peru; 58 Russian Institute of Continuous Education in Forestry, Institutskaya 17, Pushkino, 141200 Russia; 59 Institute for Evolutionary Ecology of the National Academy of Sciences of Ukraine, Lebedev 37, Kyiv, 03143 Ukraine; 60 University of Oregon, 1585 E 13th Ave, Eugene, OR 97403 USA; 61 Forest Management in Bolivia, Sacta, Bolivia; 62 FRIM Forest Reserach Institute of Malaysia, 52109 Kepong, Selangor, Kuala Lumpur Malaysia; 63 Hiroshima University, 1-7-1 Kagamiyama, Higashi-Hiroshima, Hiroshima 739-8521 Japan; 64 Center for Agricultural research in Suriname, CELOS, 1914 Paramaribo, Suriname; 65 Nicholas School of the Environment, Duke University, P.O. Box 90328, Durham, NC 27708 USA; 66 IIC, The Iwokrama International Centre for Rain Forest Conservation and Development, 77 High Street, Georgetown, Guyana; 67 Cibodas Botanic Gardens - Indonesian Institute of Sciences (LIPI), Jl. Kebun Raya Cibodas, Cipanas, Cianjur, 43253 Indonesia; 68 Museu Universitário, Universidade Federal do Acre, BR 364, Km 04 - Distrito Industrial, Rio Branco, 69915-559 Brazil; 69 Guyana Forestry Commission, 1 Water Street, Kingston Georgetown, Guyana; 70 Plant Systematic and Ecology Laboratory, University of Yaoundé I, P.O. Box 047, Yaounde, Cameroon; 71 Bioversity international, P.O. Box 2008, Messa, Yaoundé Cameroun; 72 School of Natural Sciences, Bangor University, Thoday Building. Deiniol Rd, Bangor, LL57 2UW United Kingdom; 73 Siberian Federal University, Svobodnyy Ave, 79, Krasnoyarsk, 660041 Russia; 74 Department of Forest Sciences, Luiz de Queiroz College of Agriculture, University of Sao Paolo, PO Box 9, Av. Pádua Dias, 11, Piracicaba, São Paulo 13418-900 Brazil; 75 State Nature Reserve Denezhkin Kamen, Lenina, 6, Sverdlovsk reg, Severouralsk, 624480 Russia; 76 International Center for Tropical Botany, Department of Biological Sciences, Florida International University, 11200 S.W. 8th Street, Miami, 33199 FL USA; 77 Universidad Autónoma del Beni, Riberalta, Bolivia; 78 Department of Microbiology and Ecosystem Science, Division of Terrestrial Ecosystem research, University of Vienna, Althanstrasse 14, Vienna, A-1090 Austria; 79 New Zealand Forest Research Institute (Scion) Te Papa Tipu Innovation Park, 49 Sala Street, Rotorua, 3046 New Zealand; 80 Unaffiliated (retired), Sommersbergseestrasse 291, Bad Aussee, 8990 Austria; 81 W.R.T College of Agriculture and Forestry, University of Liberia, Capitol Hill, Monrovia 9020 Liberia; 82 FRIM Forest Research Institute of Malaysia, 52109 Kepong, Selangor, Kuala Lumpur Malaysia; 83 Department Foresterie et Environnement (DFR FOREN), Institut National Polytechnique Félix Houphouët-Boigny, INP-HB, Yamoussoukro, BP 2661 Côte d’Ivoire; 84 Reshetnev Siberian state university of science and technology, pr. Mira 82, Krasnoyarsk, 660049 Russia

**Keywords:** Biogeography, Forest ecology, Forest ecology

## Abstract

Forest biomass is an essential indicator for monitoring the Earth’s ecosystems and climate. It is a critical input to greenhouse gas accounting, estimation of carbon losses and forest degradation, assessment of renewable energy potential, and for developing climate change mitigation policies such as REDD+, among others. Wall-to-wall mapping of aboveground biomass (AGB) is now possible with satellite remote sensing (RS). However, RS methods require extant, up-to-date, reliable, representative and comparable *in situ* data for calibration and validation. Here, we present the Forest Observation System (FOS) initiative, an international cooperation to establish and maintain a global *in situ* forest biomass database. AGB and canopy height estimates with their associated uncertainties are derived at a 0.25 ha scale from field measurements made in permanent research plots across the world’s forests. All plot estimates are geolocated and have a size that allows for direct comparison with many RS measurements. The FOS offers the potential to improve the accuracy of RS-based biomass products while developing new synergies between the RS and ground-based ecosystem research communities.

## Abstract (summary)

**Table:** 

Measurement(s)	above-ground biomass • organic material
Technology Type(s)	tree species census • measurement method
Factor Type(s)	geographic location • tree species
Sample Characteristic - Environment	forest biome
Sample Characteristic - Location	Earth (planet)

Machine-accessible metadata file describing the reported data: 10.6084/m9.figshare.9850571

## Background & Summary

Global estimates of forest height, aboveground biomass (AGB) and changes over space and time are needed as both essential climate variables^1^ and essential biodiversity variables^2^, and to support international policy initiatives such as REDD+ ^3^. Several space-borne missions to assess forest structure and functioning, including BIOMASS (ESA), ALOS PALSAR (JAXA), GEDI (NASA) and NISAR (NASA-ISRO), will be operational in the coming years. These missions require ground-based estimates for algorithm calibration and product validation. For instance, high-quality, standardized measurements of forest biomass and height are critical for improving the accuracy of products derived from space-borne instruments. Furthermore, ensuring that different missions have access to the same set of high-quality standardized measurements for calibration and validation should vastly help improve comparability and confidence in future remote sensing (RS) products.

Remote Sensing users typically have different product requirements compared to those of the ecological and forestry communities. Namely, RS users often (1) need access to AGB estimates at the pixel level, while ecologists and foresters produce area-based estimates derived from individual trees measurements. RS users typically (2) need products at a consistent spatial resolution, while a variety of plot sizes and shapes have been adopted by ecologists and foresters. Finally, RS users (3) require AGB to be computed via globally and regionally consistent routines, while various approaches have been developed to derive AGB estimates from tree measurements. These communities also operate differently from a funding perspective. Most notably, recurrent investments are needed to maintain permanent forest plots – including censuses that temporally match RS data collection – and to ensure field and botanical staff are paid and trained, without whom the data would not be collected. In contrast, RS users typically access data provided by space-borne missions that have already been funded. Despite these differences, there is a clear need to share existing data sets for the benefit of both communities.

The Forest Observation System – FOS (http://forest-observation-system.net/) – is an international, collaborative initiative that aims to establish a global *in situ* forest AGB database to support Earth Observation (EO) and to encourage investment in relevant field-based measurements and research^4^. The FOS enables access to high-quality field data by partnering with some of the most well-established teams and networks responsible for managing permanent forest plots globally. In doing so, FOS is benefiting both the RS and ecological/forestry communities while facilitating positive interactions between them.

To this end, the FOS project has established a data sharing policy and framework that seeks to overcome existing barriers between data providers and users. For example, data made available on the FOS website are plot-aggregated (i.e., stand AGB, canopy height, etc.), while the underlying original tree-by-tree data are managed by participating ecological networks. To ensure that estimates added to the FOS are robust and consistent, a freely downloadable BIOMASS R-package^5^ has been upgraded, which makes the procedure for computing plot AGB estimates from tropical forest inventories transparent, standardized and reproducible. There are developments underway to make the package usable for any forest type, including boreal and temperate ecosystems. This work has been complemented by the definition of a set of technical requirements and standards aimed at ensuring data comparability^4^.

The FOS currently hosts aggregate data from plots contributed by several existing networks, including: the network of the Center for Tropical Forest Science – Forest Global Earth Observatory (CTFS-ForestGEO)^6^, the RAINFOR^7^, AfriTRON^8^ and T-FORCES^9^ (curated on the ForestPlots.net platform)^10^, the IIASA network^11,12^, the Tropical Managed Forests Observatory (TmFO)^13^ and AusCover^14^. These international collaborations have already (i) invested in establishing permanent sampling plots; (ii) proposed robust protocols for accurate tree mapping and measurement, which are largely standardized across networks; (iii) monitored existing plots repeatedly; and (iv) established databases with particular emphasis on data quality control^10,15^. As the FOS is an open initiative, additional networks (e.g., GFBI^16^) and teams that comply with the aforementioned criteria are welcome to join in the future.

The data presented here have been partly published before^17–21^, but never in such a unified and comprehensive manner. Results based on some of the plots presented here have impacted a wide range of scientific fields, including tropical forest ecology^22–26^, drought sensitivity of forests^19,27–29^, tree allometry^30–33^, carbon cycles^21,34–36^, remote sensing^18,37–39^, climate change^8,40–43^, biodiversity^44–47^, diversity-carbon relationships^48,49^ and historical forest use^50,51^, among others.

**Fig. 1:**
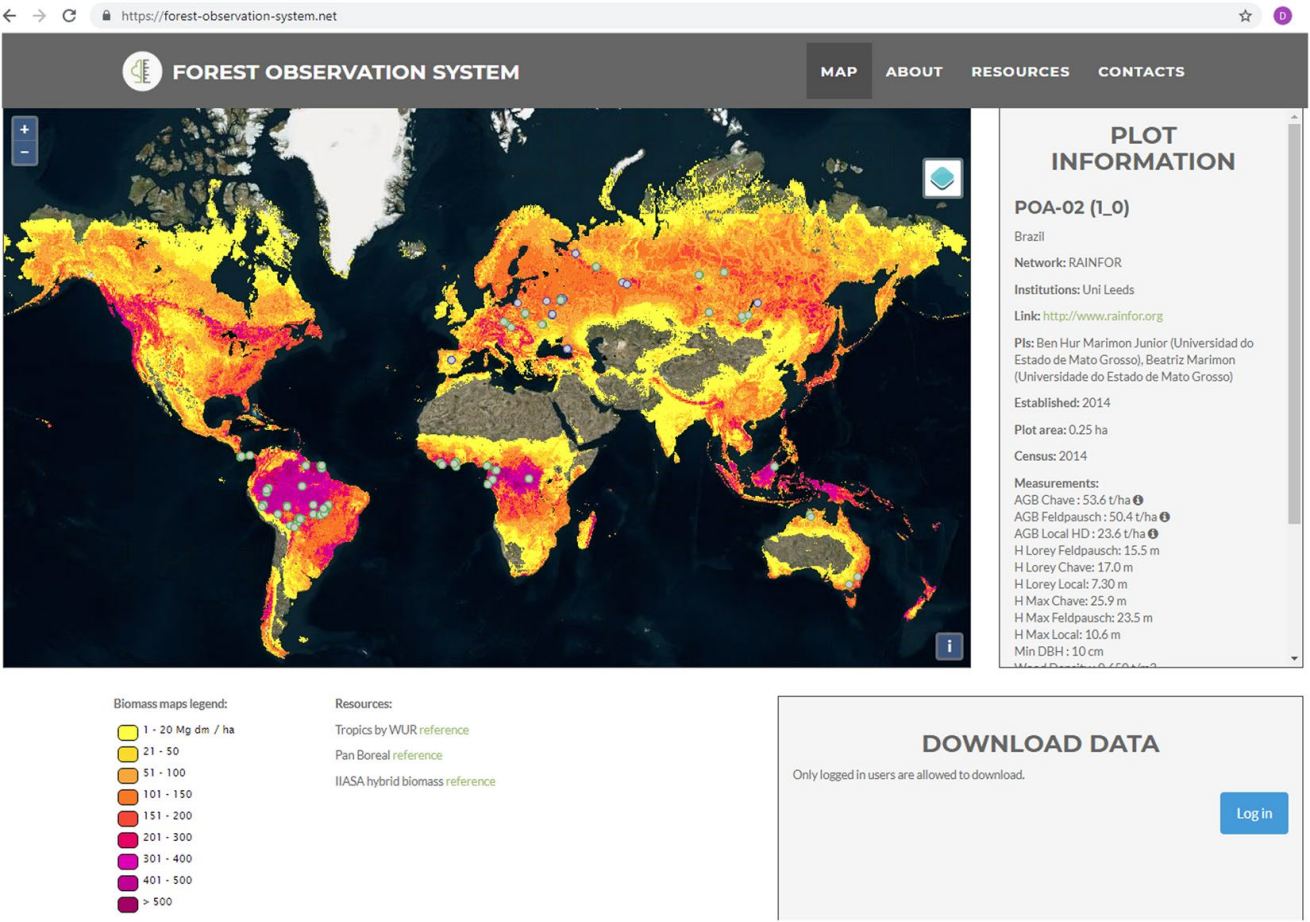
The Forest-Observation-System.net web portal.

The online database (http://forest-observation-system.net/) provides open access to the canopy height and biomass estimates as well as information about the plot PIs who have granted access to the data (see Fig. 1 below).

## Methods

Within the sample plots, every stem above a defined threshold in diameter at breast height (DBH, usually 1, 5, 7 or 10 cm) was taxonomically identified and the DBH measured, avoiding any buttresses or deformities. In most plots, tree height was measured for a subset of trees that are representative of different diameter classes and tree species in order to develop site-specific height-diameter regression equations. Based on an analysis using the tropical forest plot data, as few as 40 tree height observations are sufficient for characterizing this relationship if stratified by diameter^22^.

All the data presented here were collected from permanent forest sample plots with known locations; accurate coordinates (with an error of less than 30 meters) have been either delivered to the FOS or will be recorded during the next census. Plot sizes are typically 1 ha in area (i.e., the median), but they can vary from 0.25 ha to 50 ha. Large plots are subdivided into 0.25 ha, i.e., 50 × 50 m sub-plots. The FOS consortium made the decision to consider only relatively large and permanent plots in order to reduce errors in georeferencing and to decrease the variability in the measured parameters. Recent research has quantified the effect of spatial resolution on the uncertainties in the AGB estimates, with sampling error dropping from 46.3% for 0.1 ha plots, to 26% and 16.5% for 0.25 ha and 1 ha plots, respectively^52^. Scaling up from the plot to the landscape level using lidar-derived metrics, studies have shown decreases in the RMSE for the AGB-lidar models, from 70–90 to 36–51 Mg AGB per ha, when increasing the plot size from 0.25 ha to 1 ha^17,53^. Clearly there are always size-effort tradeoffs, e.g., smaller plots would permit greater replication, but by focusing on larger plots that are also permanent, FOS has chosen to focus its efforts on a smaller but high-quality set of plots. Our approach, therefore, excludes the possibility of using databases of smaller plots such as those found in national forest inventories.

**Fig. 2:**
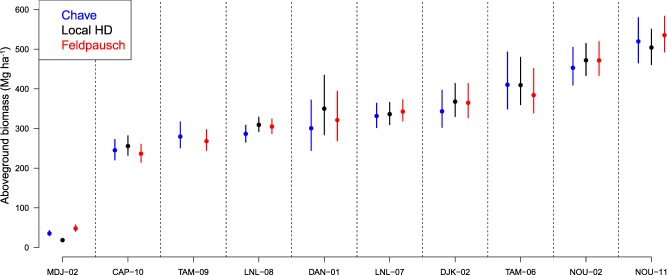
An example of the AGB estimation with the BIOMASS R-package. MDJ-02, CAP-10 and other indexes on the horizontal axis are Plot IDs. The vertical axis is AGB in Mg ha^−1^ and the error bar represents the credibility interval at 95% of the stand AGB value following error propagation.

AGB and associated uncertainties were obtained using a standardized procedure implemented in the BIOMASS R-package^5^. For the sake of standardization, we systematically considered only trees having a diameter ≥10 cm (or a 5 cm threshold in the case where these trees contribute substantially (>5%) to the total AGB, e.g., in savannas). Taxonomy was first checked using the Taxonomic Name Resolution Service, which in turn served to assign a wood density value to each tree using the Global Wood Density Database (GWDD) as a reference^54,55^. Species- or genus-level averages were assigned when possible and, if not, the plot-level mean wood density was assigned to each tree species with no known wood density. Tree height was estimated in three different ways. First, when available, subsets of tree height measurements were used to build plot-specific height-diameter relationships, assuming a three-parameter Weibull model^5^ or a two-parameter Michaelis-Menten model, whichever provided the lowest prediction error. Secondly, the regional height-diameter models proposed by Feldpausch *et al*.^31^ were used to infer tree height. Finally, height was implicitly taken into consideration in the AGB calculation through the use of the bioclimatic predictor E proposed by Chave *et al*.^30^. Equation 7 of Chave *et al*.^30^ was used in this case while the generalized allometric model equation 4 was used otherwise (where heights were derived from local or Feldpausch height-diameter relationships). Among the three approaches, the use of a local HD model is the most accurate. However, local height measurements are not systematically available for all plots. The Chave *et al*. (2014) and Feldpausch *et al*. (2012) approaches are both an alternative to the use of a local HD model but independent validation (e.g., Fig. 2) has shown that their relative performance varies among locations. Thus, the most conservative approach is to provide the three estimates so that the uncertainty associated with the HD relationship can be assessed.

Errors associated with each of these steps (i.e., DBH measurement, wood density, tree height) were propagated through a Monte Carlo scheme to provide mean AGB estimates with associated credibility intervals (Fig. 2).

Boreal and temperate plots (representing 11% of the total number of sub-plots) were processed manually using similar steps. Species-specific allometric equations^56^ allowed the stem volume to be estimated based on the height and DBH measurements. Biomass conversion and expansion factors^57^ were used to estimate AGB from the stem volume taking the tree age, site index and stocking into account. The next version of the BIOMASS R-package will be capable of processing boreal and temperate data in addition to tropical.

## Data Records

**Fig. 3:**
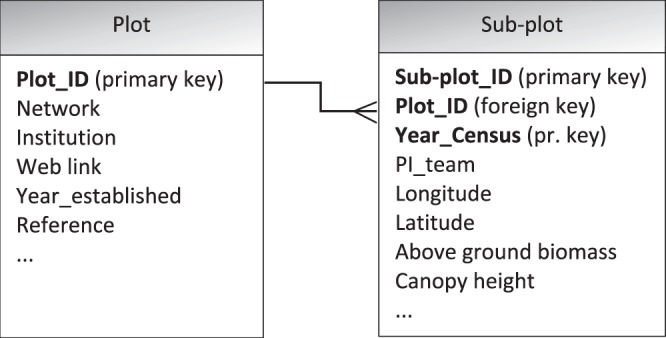
The database structure of the plot information.

The data in FOS^58^ are organized in a hierarchical structure (Fig. 3). The **Plot** description includes a link to the institution and network. The central part of the database is the **Sub-plot** table, where geolocation, the date of the census, the people who manage the specific plots, the AGB and the canopy height are stored.

The FOS does not store individual tree-level information, only plot-level aggregates. Users interested in tree-level information can contact the contributing networks or the plot PIs using the links provided in the Plot table.

The details of the fields found in the two linked tables of Fig. 3 are provided below.

Plot description

- Plot_ID – unique plot ID
- Country_Name – Name of the country
- Network – the name of the network (e.g., RAINFOR)
- Institution – the institution that carried out the measurements
- Link – web link to the data provider
- Year_established – the year when the plot was established
- Reference – a reference to the publications
- Other_measurements – list of parameters measured on the plot
- Biomass_processing_protocol – file name of the biomass processing protocol (available at Data Package 1), which contains the R code, the variables assigned and the intermediate results.

Sub-plot description

- Sub-plot_ID – unique sub-plot ID
- Plot_ID – link to the **Plot description** table
- Year_census – year of the census
- PI_team – List of Principal Investigator(s)
- Lat_cnt – Latitude of the center of the plot
- Long_cnt – Longitude of the center of the plot
- Altitude (m a.s.l.)
- Slope (degree)
- Plot_area (ha)
- Plot_shape (e.g., rectangle, circle, plus dimensions)
- Forest_status – forest description, including age, successional stage, disturbances, etc.
- Min_DBH – Minimum diameter of trees at breast height included in the census (cm)
- H_Lorey – Lorey’s height, DBH-weighted mean tree height (m)
  - H_lor_ local – mean height estimated from local H = f(DBH) curve (m)
  - H_lor_ Chave – mean height estimated from the curve by Chave^30^ (m)
  - H_lor_ Feldpausch – mean height estimated from the curve by Feldpausch^31^ (m)
- H_max – height of the tallest tree (m)
  - H_max_ local – tallest tree measured or estimated from local H = f(DBH) curve (m)
  - H_max_ Chave – maximum height estimated from the curve by Chave (m)
  - H_max_ Feldpausch – maximum height estimated from the curve by Feldpausch (m)
- AGB – Above ground biomass (Mg ha^−1^)
  - AGB_local – aboveground biomass (Mg ha^−1^) estimated using local equations or equation 4 in Chave^30^ with wood density, DBH and H derived from local height-diameter relationships.
    - Cred_2.5 – lower bound of 95% credibility interval (Mg ha^−1^)
    - Cred_97.5– upper bound of 95% credibility interval (Mg ha^−1^)
  - AGB_Feldpausch – AGB (Mg ha^−1^) using equation 4 in Chave^30^ with wood density, DBH and H derived from Feldpausch^31^ height-diameter relationship.
    - Cred_2.5 – lower bound of 95% credibility interval (Mg ha^−1^)
    - Cred_97.5 – upper bound of 95% credibility interval (Mg ha^−1^)
  - AGB_Chave – aboveground biomass (in Mg ha^−1^) estimated using equation 7 in Chave^30^ with wood density, DBH and H implicitly taken into consideration through the use of the bioclimatic predictor E
    - Cred_2.5 – lower bound of 95% credibility interval (Mg ha^−1^)
    - Cred_97.5 – upper bound of 95% credibility interval (Mg ha^−1^)
- Wood_density - mean wood density of the trees (g cm^−3^)
- GSV – growing stock volume (m^3^ ha^−1^)
- BA – basal area (m^2^ ha^−1^)
- Ndens – number of trees per hectare

Note that we have merged the Plot and Sub-plot tables in the data package associated with this paper^58^ for the user’s convenience.

## Technical Validation

The key predictive variables of AGB are tree dimensions (primarily diameter and height) and taxonomic identity, which is responsible for explaining most tree-to-tree variations through interspecific wood density variations^59^. The procedures for ensuring the quality of the data collected are as follows:

1. *On-site measurement accuracy*. To ensure diameter accuracy and consistency among and within censuses, field teams follow standard forest inventory protocols for the correct choice of the Point of measurement (POM). For example, the RAINFOR protocol for tropical forests^60^ records each POM by painting the location on each tree to ensure that subsequent measurements can be performed at the same point. For tree height, the consistency of the height measurement is ensured by having a designated, trained operator who works at multiple sites using the same instrument. At some sites, double measurements of height (from different positions) have been carried out, and mean values have been used as the height of the individual trees. For species identification, the reliability in highly diverse tropical plots is important; hence, the tree and plot AGB is estimated by taking the species-level variability in wood density into account^61^. This is supported by collecting botanical vouchers from every taxon (or potential taxon) in the field. In many cases, these vouchers have been deposited in recognized regional herbaria, identified by botanical experts, and where possible, made available electronically (e.g., via ForestPlots.net). However, voucher collection is not currently a standard protocol for every plot in the FOS.
2. *Multiple censusing*. By working primarily with re-censused permanent plots rather than single census plots, we have ensured that the uncertainties are reduced because almost every tree has been measured at least twice by the time of the focal census, thus providing the opportunity to correct any errors that may have been made previously, through the identification of spurious values. Repeat censuses also provide more opportunities to improve species identification by increasing the chance of encountering fertile material (see the next step).
3. *Post fieldwork data processing*, e.g., by identifying trees to species level. Species identification can be extremely challenging in tropical forests due to their diversity and the fact that most trees lack flowers or fruits when inventoried. Botanical identity is a key control on the AGB through its effect on wood density. To explore the reliability of identification in some of the most diverse RAINFOR sites in western Amazonia, PIs have separated the tree species assemblages into several larger taxonomic groups. As reported by Baker *et al*.^62^, taxonomic specialists for each group have then assessed the accuracy of the species identifications of the herbarium collections using 18 different botanists across 60 plots during the past 30 years. Overall, even in taxonomically difficult groups where species are often very rare, 75% of tree species were correctly identified.
4. *Common protocols for potential error detection*. These protocols have been developed by contributing networks, e.g., by flagging trees for attention that have declined by more than 5 mm in diameter. This allows trees to be detected that have shrunk between two censuses, and whether that individual is dead/rotten. Potential issues are flagged in order to be checked against existing field notes, and during the following census. Thus, as mentioned previously, repeat censuses provide more opportunities to improve data quality as compared to single-census plots.
5. *Within-network collaboration*. Data quality is further enhanced through the exchange of ideas between experts at different sites and between nations, through the use of common data analysis protocols (i.e., allometric equations, R packages, etc.), and by promoting shared publications.
6. *Cross-network collaboration*. In the FOS, by applying a uniform R script for data aggregation and AGB estimation, potential biases from using different height-diameter, wood density and allometric relations are strongly reduced.

The distribution of FOS plots by continent is presented in Table 1. Africa, Europe and South America are represented by similar numbers of locations (i.e., 62–80 plots) and contribute more than 80% of the plots at the time of publication, but in terms of coverage, South America alone comprises 49% of the forest area covered.

**Table 1:** Distribution of records by continents (as of December 2018).

Continent/regions	Number of plots	Number of sub-plots	Area (ha)
Africa	62	338	85
Asia	29	46	20
Australia	4	4	3
Central America	21	278	69
Europe	80	146	42
South America	78	833	209
Total	274	1645	428

The IIASA network provides the highest number of plot locations to FOS (Table 2), while the TmFO network contributes the most in terms of areal coverage.

**Table 2:** The distribution of records by participating networks (as of December 2018).

Network	Number of plots	Number of sub-plots	Area, ha
AfriTRON	46	178	45
AusCover	4	4	3
CTFS-ForestGEO	2	300	75
IIASA	126	258	78
RAINFOR	52	288	72
T-Forces	3	12	3
TmFO	17	500	125
Unaffiliated to network	24	105	27
Total	274	1645	428

The range of values of major forest parameters represented in the FOS database is shown in Table 3. The maximum AGB value (918 Mg ha^−1^) and canopy height (41.7 m) at a 0.25 ha sub-plot were recorded in Lopé, Gabon. Some savannah sub-plots (e.g., in Gabon) have a few or no trees >5 cm dbh, which leads to low or no biomass estimation. The tallest trees (60.1 m) was found in Costa Rica and the maximum basal area (85.6 m^2^ ha^−1^) was found in the Caucasus, Russia.

**Table 3:** The range of major forest parameters in the FOS database (as of December 2018).

Parameters	min	Max	Median
Latitude	−36.52	64.51	5.26
Longitude	−83.58	148.92	−52.92
Plot area	0.25	50	1.0
Sub-plot area	0.0625	2.89	0.25
Year established	1955	2017	2012
Year of census	1999	2018	2012
Min DBH, cm	1	10	10
Height Lorey’s, m	2.3	41.7	26.8
Height max, m	3.6	60.1	39.3
AGB, t ha^−1^	0.3	933	258
Basal area, m^−2^ ha^−1^	0.05	85.58	28.32
Tree density, trees ha^−1^	4	1800	452

Table 4 contains information about the AGB for different biomes and globally. As expected, the average AGB increases from boreal to temperate and then from temperate to tropical forests.

**Table 4:** The distribution of aboveground biomass data (t ha^−1^) by biome in the FOS database (as of December 2018).

Biome	Min	Max	Average	StdDev
Boreal	1	324	99	68
Temperate	48	609	211	75
Tropics	0.3	933	311	132
Global	0.3	933	299	133

## Usage Notes

This data set will be essential for validating and calibrating satellite observations and forest biometric models. The focus is to provide ground support for current and planned space-borne missions, such as NASA GEDI (https://gedi.umd.edu/), NASA-ISRO NISAR (https://nisar.jpl.nasa.gov/), JAXA ALOS PALSAR (http://global.jaxa.jp/projects/sat/alos/) and ESA BIOMASS (https://earth.esa.int/web/guest/missions/esa-future-missions/biomass), which are aimed at retrieving forest structure parameters such as forest height and biomass.

At this stage, we are making no claims regarding the statistical robustness of the FOS data set for global or regional biomass estimations. Instead our aim is to present uniformly processed data on forest biomass from available locations (see Table 1). One of the main goals of the FOS is to highlight gaps in the observations.

Using sub-plot data for validation of RS data might lead to spatial autocorrelation problems so possible solutions would be to use a plot average, use only values from the plot or test for the presence of spatial autocorrelation.

This data package contains geographical coordinates rounded to 2 digits after decimal point (up to 1 km at equator). The most up-to-date extended data set with accurate geolocation is available in the FOS portal: https://forest-observation-system.net/

The FOS initiative depends on the contributions of high-quality forest plot data from participating networks. The fair use of the data presented here requires respecting the efforts and rights of the partners and supporting the long-term future of these observational efforts. The data set will be licensed under a Creative Commons Attribution 4.0 International License (CC-BY 4.0), which means that it will be fully open even for commercial use but requires acknowledgment of the PIs and plot owners. We would also appreciate that all users of the FOS data either share their own data via the FOS, and/or commit to collaboratively funding new censuses and the expansion of existing plot networks.

## Data Availability

The BIOMASS R-package is an open source library available from the CRAN R repository. The development version is publicly available and can be found on the GitHub platform at: https://github.com/AMAP-dev/BIOMASS. Furthermore, the BIOMASS R-package is accompanied by an open access paper describing the functionality in more detail^5^.
